# The effect of gallic acid on memory and anxiety-like behaviors in rats with bile duct ligation-induced hepatic encephalopathy: Role of AMPK pathway

**DOI:** 10.22038/AJP.2022.19720

**Published:** 2022

**Authors:** Leila Jafaripour, Khadijeh Esmaeilpour, Marzieh Maneshian, Hamideh Bashiri, Mohammad Amin Rajizadeh, Hassan Ahmadvand, Majid Asadi-Shekaari

**Affiliations:** 1 *Department of Anatomical Sciences, Afzalipour Medical School, Kerman University of Medical Sciences, Kerman, Iran*; 2 *Neuroscience Research Center, Institute of Neuropharmacology, Kerman University of Medical Sciences, Kerman, Iran*; 3 *Physiology Research Center, Institute of Basic and Clinical Physiology Sciences and Department of Physiology and Pharmacology, Kerman University of Medical Sciences, Kerman, Iran*; 4 *Medical Plants and Natural Products Research Center, Hamadan University of Medical Sciences, Hamadan, Iran *; 5 *Razi Herbal Medicine Research Center, Lorestan University of Medical Sciences, Khorramabad, Iran*

**Keywords:** Gallic acid, Bile duct ligation, Hepatic encephalopathy, AMPK activation, Memory

## Abstract

**Objective:**

Hepatic encephalopathy (HE) is a serious neurological syndrome which is caused by acute and chronic liver diseases. In this study, the effect of gallic acid (GA) as an activator of AMP-activated protein kinase (AMPK) on memory and anxiety-like behaviors in rats with HE caused by bile duct ligation (BDL) was investigated.

**Materials and Methods:**

The rats were randomly divided into the following eight groups (n=7): sham; BDL; BDL+GA 20 mg/kg; BDL+GA 30 mg/kg; sham+dorsomorphin or compound C (CC) (as AMPK inhibitors); BDL+CC; BDL+GA 20 mg/kg+CC; and BDL+GA 30 mg/kg+CC. The rats received GA once daily by gavage for four weeks, and dorsomorphin 6.2 µg per rat was administered on a daily basis via bilateral intraventricular injection for four weeks. Behavioral tests including novel object recognition (NOR), open field and Morris water maze (MWM) were used to evaluate anxiety and memory in the rats.

**Results:**

Examining some parameters of NOR and MWM tests showed that memory performance was significantly reduced in the BDL versus the sham group, and in the BDL+CC versus the sham+CC group (p<0.05). GA intake improved memory in the GA-receiving groups compared with the BDL and BDL+CC groups (p<0.05). Examining some parameters of open field test showed that anxiety was significantly increased in the BDL versus the sham group, and the BDL+CC versus the sham+CC group (p<0.05). GA intake reduced anxiety in GA-receiving groups compared with the BDL+BDL+CC group (p<0.05).

**Conclusion:**

GA was effective in improving cognitive and anxiety-like behaviors through activating AMPK.

## Introduction

Hepatic encephalopathy (HE) is a serious neuropsychiatric syndrome resulted from acute and chronic liver diseases. Overt symptoms of HE include personality changes, sleep disturbances, ataxia, and asterixis as well as impairment of learning ability and memory formation, which may ultimately lead to coma and death (Braissant et al., 2019 ▶; Jefferson et al., 2020 ▶). Liver damage leads to the increase in blood ammonia and as a result, it disrupts the central nervous system and increases oxidative stress and neuroinflammation (Lu et al., 2020a ▶). 

Cholestasis causes accumulation of bile acids in the liver which leads to stimulatory effects on hepatocytes and hepatic inflammation, oxidative stress, fibrosis and hepatocyte death (Lin et al., 2017 ▶). Bile duct ligation (BDL) is a classical model that leads to biliary cirrhosis in animal models. Morphological changes of cirrhosis in this model were comparable to those observed in human biliary cirrhosis. According to the International Society for Hepatic Encephalopathy and Nitrogen Metabolism (ISHEN, the BDL model leads to cholestatic liver diseases or chronic liver failure (CLF), resulting in the HE manifestations (Butterworth et al., 2009 ▶). HE following cholestasis in the BDL model in rats causes cognitive disorder, memory impairments, anxiety-like behaviors and exploratory behaviors (Esfahani and Zarrindast, 2021 ▶). 

Adenosine monophosphate-activated kinase (AMPK), which is a heterotrimeric serine/threonine kinase, is one of the key factors in protecting intracellular homoeostasis and it can be activated by increased intracellular AMP levels (Hardie et al., 2012 ▶; Heidrich et al., 2010 ▶). AMPK activates catabolic pathways that produce ATP. It not only plays a vital role in response to diverse metabolic stresses such as hypoxia, oxidative stress and inflammation, but also has a more important role than most types of stresses in maintaining intracellular homoeostasis (Hardie et al., 2012 ▶; Heidrich et al., 2010 ▶). AMPK in the hippocampus is activated by cyclosporine A and a ketogenic diet. Cyclosporine A activates the downstream kinases involved in the lipid metabolism and phosphorylation of acetyl-CoA carboxylase (ACC) which is induced by the ketogenic diet. The AMPK/ACC pathway may be new target for treating neurodegenerative diseases, such as Parkinson’s, Alzheimer’s and stroke (Jeon et al., 2009 ▶; Park et al., 2011 ▶). AMPK promotes neurogenesis in the hippocampus by improving mitochondrial metabolism, thus reducing depression and anxiety-like behaviors and improving memory and learning (Finley, 2018 ▶; Sun et al., 2020 ▶). Compound C (6[4‑(2‑piperidin‑1‑yl‑ethoxy)‑phenyl] 3‑pyridin‑4‑yl‑pyrazolo[1,5‑a] pyrimidine) or dorsomorphin is a small molecule that interferes with AMPK metabolic regulation and is used as an AMPK inhibitor (Chuang et al., 2021 ▶). Dorsomorphin causes memory impairment following the AMPK inhibition (Rashtiani et al., 2021b ▶).

Gallic acid (3, 4, 5-trihydroxybenzoic acid, GA) is a natural polyphenol with antioxidant and anti-inflammatory properties (Eslamifar et al., 2021 ▶). GA activates AMPK pathways through increasing antioxidant capacity, and increasing mitochondrial mass and fatty acid metabolism, which can be effective in ameliorating inflammatory diseases, metabolic syndromes and atherosclerosis (Doan et al., 2015 ▶; Tanaka et al., 2020 ▶). 

Due to the complexity of the factors that play a role in the HE development, nowadays, drug therapies and therapies combined with diets appropriate for individuals, especially diets rich in antioxidants, can have better results. Therefore, the goal of this study is to explore how GA with its antioxidant properties can activate AMPK. Whether it can reduce memory disorders and anxiety-like behaviors in HE due to BDL in an animal model or not. 

## Materials and Methods

**Chemicals**

All the chemicals used in the study were of analytical grade. GA (CAS Number: 149-91-7) and dorsomorphin (CAS Number: 66405-64-3) were purchased from Sigma Chemical of Taufkirchen, Germany.

**Experimental procedures**

**Animals and treatment plan**

Fifty-six male Wistar rats weighing 220-250 g were purchased from Neuroscience Research Center, Kerman University of Medical Sciences, Kerman, Iran. They were allowed to acclimate to the laboratory conditions for one week before beginning the experiment. The animals were housed in Plexiglas boxes with sawdust bedding in a 12:12-hour day/night cycle, at 22±1ºC. All the experimental stages were approved by Institutional Animal Ethics Committee, Kerman University of Medical Sciences, Kerman, Iran (ethical number: IR.KMU.REC.1399.470). The rats were randomly divided into eight groups (n=7) as follows: 

Sham: Rats underwent laparotomy without BDL surgery. 

BDL: Rats underwent BDL surgery. 

BDL+GA 20 mg/kg: Rats underwent BDL surgery and received GA (Abdel-Moneim et al., 2017 ▶)

BDL+GA 30 mg/kg: Rats underwent BDL surgery and received GA (Mansouri et al., 2013 ▶). 

Sham+CC: Rats underwent laparotomy and received dorsomorphin or compound C (CC). 

BDL+CC: Rats underwent BDL surgery and received dorsomorphin. 

BDL+GA 20 mg/kg+CC: Rats underwent BDL surgery and received GA and dorsomorphin. 

BDL+GA 30 mg/kg+CC: Rats underwent BDL surgery and received GA and dorsomorphin. 

GA was administered once by gavage for four weeks the day after BDL surgery. Other groups received normal saline by gavage on the daily basis for 4 weeks. 

Dorsomorphin (6.2 µg) was administered through bilateral intraventriclar (i.c.v) injection for four weeks for all the receiving groups (Zarei et al., 2019 ▶). Other groups received normal saline through bilateral intraventriclar (i.c.v) injection on the daily basis for 4 weeks.

Dorsomorphin was dissolved in 5% DMSO in normal saline and GA was dissolved in normal saline.

**Rat brain in stereotaxic coordinates**

The animals were anesthetized by intraperitoneal (i.p.) injection of a combination of ketamine (87.5 mg/kg) and xylazine (12 mg/kg). After shaving their scalp by clippers, the rats were placed in the stereotaxic instrument and an incision was made on the mid line of the scalp. The cannulas were inserted into the ventricle for i.c.v injection (coordinates: ±1.3 mm lateral to the midline, 0.8 mm posterior to the bregma and 3.4 mm ventral to the surface of the skull). Then, a small hole was drilled by a micro dental drill in the skull. Cannulas were bilaterally fixed to the parietal skull by two small screws and dental acrylic cement (Zarei et al., 2019 ▶). 

**BDL surgery**

Each rat was anesthetized by ketamine (87.5 mg/kg) and xylazine (12 mg/kg, i. p.). 

After shaving the hair and disinfecting the site, an incision was made in the midline of the abdomen and the liver was retracted gently to identify the common bile duct. The common bile duct was carefully separated and double ligated with a 4–0 silk suture and cut between these two ligatures. Then, the surgical incision was sutured in two layers. In the sham group, abdominal incision was performed, but the common bile duct was not ligated. All the rats were kept and monitored for four weeks after BDL surgery (Golshani et al., 2019 ▶).

**Novel object recognition test**

The novel object recognition (NOR) test is used to assess recognition memory and learning in the animal models of neurological disorders. All the animals were habituated to a plastic cage (57×57× 40 cm) for 10 min. The next day (the training phase), each animal was allowed to freely explore two similar objects. The objects were identical both in height and mass, but different in appearance. A training session was offered in an identical location inside the box for 5 min. Memory retention was measured throughout the testing session, 15 min after the education session. In the testing phase, to prevent the animals from being naturally attracted to a place in the box, the object position was changed randomly. The animals were allowed to explore the box for 3 min.

NOR performance was determined by the discrimination index (DI). DI in the training phase indicates the exploration time for one of the familiar objects (T _familiar_) to the total time. DI in the testing phase indicates the exploration time for the novel object (T _novel_) to the total time. The total time is the exploration time of both objects in the training and testing phases (T_total_) (Joushi et al., 2021 ▶). 

DI _training phase_ = T _familiar _/ T_total_

DI _test phase_ = T _novel _/ T_total_

**Open field test**

The open field test is a well-known protocol used to determine exploratory behavior, general locomotor activity and anxiety in animals such as rats, mice and rabbits. 

In the present study, the rats were located in a large square chamber (80×80× 50 cm) made of Plexiglas. Each animal was slowly placed in the center of the arena. It was allowed to move freely for 5 min in the arena. The digital video camera in the room recorded all the behaviors of the rat and the obtained information was analyzed by Ethovision software. This test examined parameters such as total distance moved (TDM, in cm), time duration of movement (M, in sec), cumulative duration in the center zone (CDCZ, in sec), cumulative duration in the peripheral zone (CDPZ, in sec) and numbers of rearing (R) and grooming (G) (Safa et al., 2020 ▶).

**Morris water maze test**

The Morris water maze (MWM) is a test that is extensively used to investigate spatial memory and learning in animal models. In this test, the rats were placed in a round black pool of water, 160 cm in diameter and 80 cm in height. The pool was filled with water to the depth of 40 cm at the room temperature (26°C) and was divided into equal quarters; the starting points of the quarters were called N, S, E and W. A platform with the diameter of 10 cm was placed in the pool in such a way that could not be seen by the animals. The experiment was performed in a dimly lit room with varied and tortuous images attached to different parts of the wall around the maze. All the experimental groups were tested during the lighting time between 9:00 am and 7:00 pm. All the testing stages were registered by the Smart video tracking system and the rats could be followed on the screen of a computer. Each animal completed three blocks of training that were separated by a period of 30 min resting. Each block consisted of four trials with 60-sec intervals. In each trial, the animals were placed in the water pool randomly from each of the four quadrants of the maze. During the acquisition, the platform's location remained constant and the rats were allowed to search the pool and find the platform for 60 sec. If an animal could not find the platform in 60 sec, it was guided toward the platform by the experimenter. The velocity, time spent to find the platform and distance moved were calculated and analyzed. Spatial memory was tested 24 hr after the final training test. In this test, the platform was removed and the animal was allowed to swim in the pool for 60 sec. The time spent and the distance moved in the target quadrant were analyzed as memory indices (Rajizadeh et al., 2019 ▶).

**Statistical analysis**

In this study, SPSS 23 software was used to analyze the data. After testing the normality of data using Shapiro-Wilk test, they were analyzed using analysis of variance (ANOVA) or nonparametric tests. In the open field test, velocity and distance traveled were compared using one-way ANOVA. Other parameters were analyzed by nonparametric tests (Kruskal–Wallis test) and the data are expressed as median (min-max). The discrimination index in the training and testing in NOR test was analyzed by one-way ANOVA. In acquisition trail in the MWM test, the parameters were analyzed using two-way ANOVA and Tukey post hoc test for making comparison between the two groups. In probe trail in the MWM test, the parameters were analyzed using one-way ANOVA. The data are expressed as mean±standard error of the mean (SEM). p-values equal to or lower than 0.05 were considered significant.

## Results

**Effect of GA on NOR in BDL rats**

**Figure 1:**
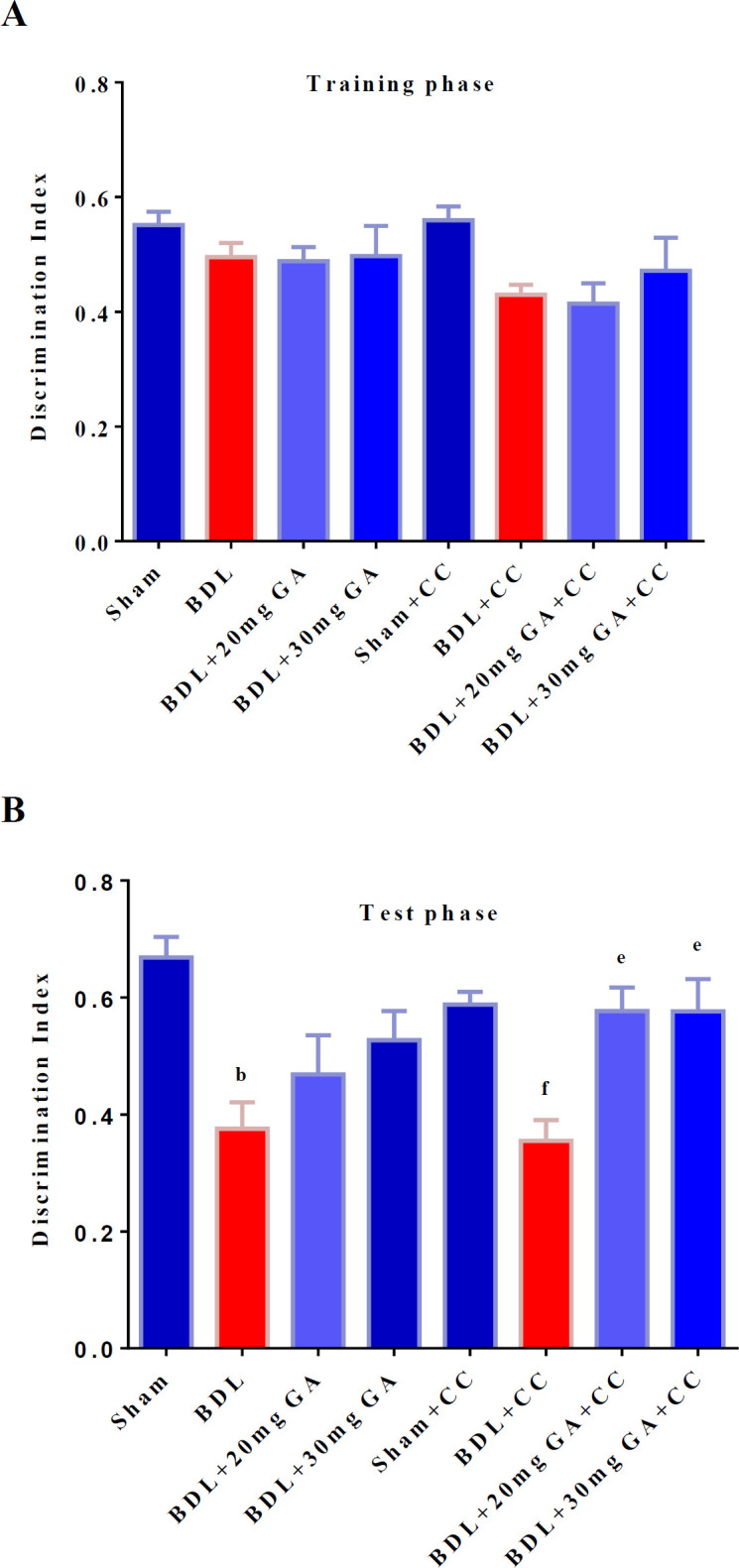
Short-term memory performance in the novel object test after BDL and four weeks of GA and dorsomorphin intake. The discrimination index in the training and testing phases was analyzed by one-way ANOVA. a shows a significant difference from the BDL group. b shows a significant difference from the sham group. c shows a significant difference from the BDL+GA 20 mg/kg group. d shows a significant difference from the BDL+GA 30 mg/kg group. e shows a significant difference from the BDL+CC group. f shows a significant difference from the sham+CC group

One-way ANOVA did not show a significant difference among the groups in terms of the discrimination index in the training phase [F (7,48) =2.19, p=0.06] (Figure 1A). 

In this study, analyzing the discrimination index in the testing phase using one-way ANOVA revealed significant differences among the groups [F (7, 47) =5.56, p<0.0001]. Analysis by Tukey’s *post-hoc* test showed that DI was significantly decreased in the BDL group compared with the sham group (p=0.001). The BDL+GA 20 mg/kg group demonstrated no significant difference from the BDL group in the discrimination index in the testing phase (p=0.052). The BDL+GA 30 mg/kg group was not significantly different from the BDL group in the discrimination index in the testing phase (p=0.071). 

In this study, analysis by Tukey’s *post-hoc* test showed that DI was significantly decreased in the BDL+CC group compared with the sham+CC group (p=0.029). The BDL+GA 20 mg/kg+CC group showed a significant increase in the discrimination index in the testing phase compared with the BDL+CC group (p=0.032). The BDL+GA 30 mg/kg+CC group had a significant increase in the discrimination index in the testing phase compared with the BDL+CC group (p=0.045) (Figure 1B).

**Effect of GA on open field test in BDL rats**

**Figure 2:**
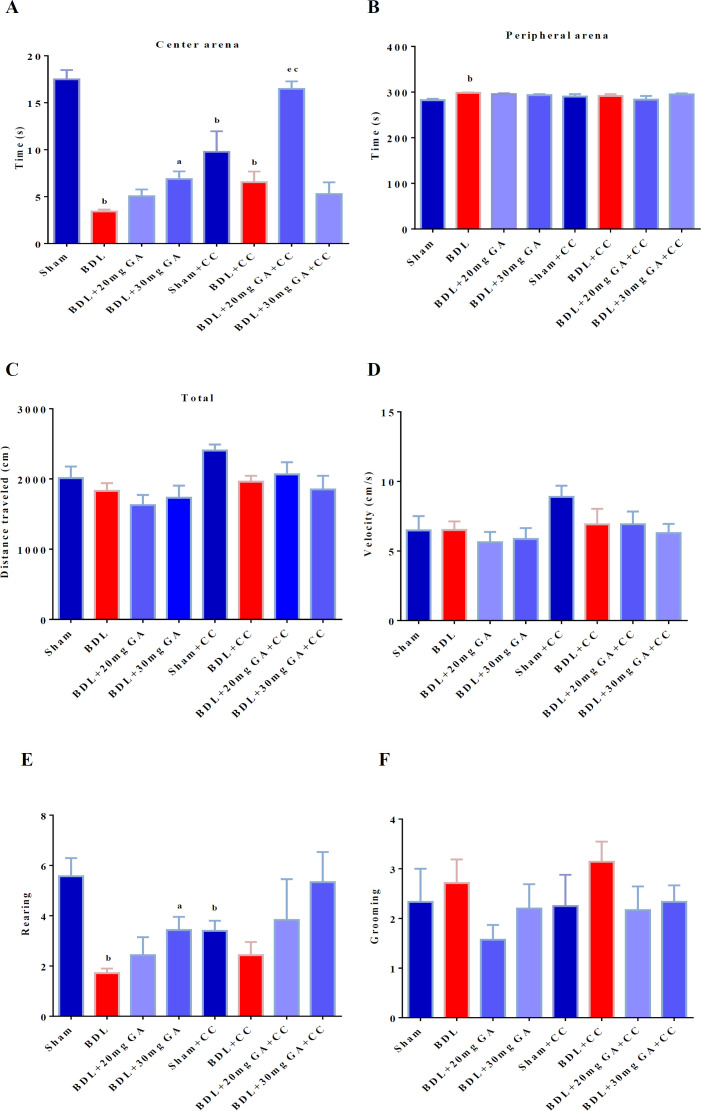
Measurement of general locomotor activity and evaluation of the anxiety level by the open field test after BDL and four weeks of GA and dorsomorphin intake. Data were analyzed by the Kruskal–Wallis test or one-way ANOVA with Tukey’s *post-hoc* test. The values are presented as mean±SEM a shows a significant difference from the BDL group. b shows a significant difference from the sham group. c shows a significant difference from the BDL+GA 20 mg/kg group. d shows a significant difference from the BDL+GA 30 mg/kg group. e shows a significant difference from the BDL+CC group. f shows a significant difference from the sham+CC group

Kruskal–Wallis test showed a significant difference among the groups in the time spent in the center zone by the rats [H (7) =34.71 (p<0.0001). The BDL group showed a significant decrease compared with the sham group (p=0.001). The BDL+GA 20 mg/kg group did not show a significant difference from the BDL group in the center zone (p=0.26). The BDL+GA 30 mg/kg group had a significant increase compared with the BDL group in the center zone (p=0.022). The BDL+CC group did not demonstrate a significant difference from the sham+CC group in the center zone (p=0.34). The BDL+GA 20 mg/kg+CC group showed a significant increase in the center zone compared with the BDL+CC group (p=0.001) and the BDL+GA 30 mg/kg +CC group had no significant difference from the BDL+CC group in the center zone (p=0.29). Moreover, the BDL+GA 20 mg/kg+CC group showed a significant increase from the BDL+GA 20 mg/kg in the center zone (p=0.001) (Figure 2A).

Kruskal–Wallis test did not show a significant difference among the groups in the duration spent in the peripheral zone by the rats [H(7) =13.06, (p=0.071)]. However, a significant difference was observed between the BDL and sham groups in duration spent in the peripheral zone (p=0.001) (Figure 2B).

One-way ANOVA showed a significant difference among the groups in the distance traveled by animals [F (7, 48) = 2.34, (p=0.04)]. The BDL group showed no significant difference from the sham group in the distance traveled by animals (p=0.98). The BDL+CC group was not significantly different from the sham+CC group in the distance traveled by animals (p= 0.48) (Figure 2C).

In this study, one-way ANOVA did not show statistically significant difference among the groups in terms of the movement velocity of the rats [F (7, 50) = 1.20, (p=0.32)] (Figure 2D).

In the present study, Kruskal–Wallis test showed a statistically significant difference among the groups in the rearing behaviors [H (7) =17.68, (p=0.014). The BDL group showed a significant decrease in the rearing behaviors compared with the sham group (p=0.002). The BDL+ GA 30 mg/kg group had a significant increase in the rearing behaviors compared with the BDL group (p=0.026). The sham+CC group showed a significant decrease in the rearing behaviors compared with the sham group (p=0.03) (Figure 2E).

Kruskal–Wallis test showed no significant difference among the groups in the grooming behavior [H(7) =9.11, (p=0.25)] (Figure 2F). 

**Effect of GA on the** **MWM test in BDL rats**

**Figure 3:**
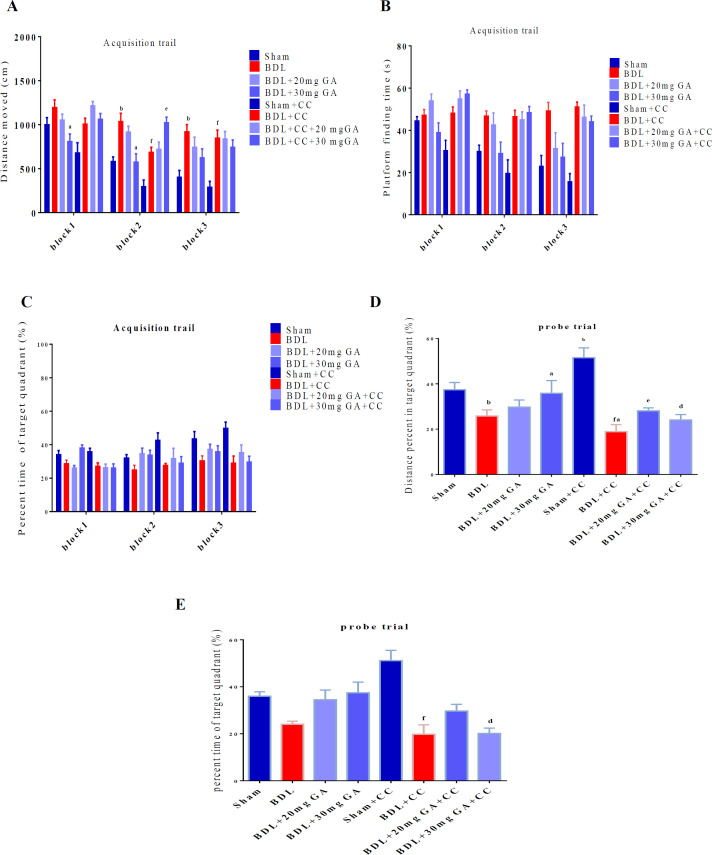
The MWM test for short-term memory after BDL and four weeks of GA and dorsomorphin intake. The data of the MWM acquisition trail were analyzed by two-way ANOVA and Tukey post hoc test for comparison between the two groups; the MWM probe trail was analyzed by one-way ANOVA. The values are presented as mean±SEM. a shows a significant difference from the BDL group. b shows a significant difference from the sham group. c shows a significant difference from the BDL+GA 20 mg/kg group. d shows a significant difference from the BDL+GA 30 mg/kg group. e shows a significant difference from the BDL+CC group. f shows a significant difference from the sham+CC group

The data on the distance moved are shown in Figure 3A. Two-way ANOVA demonstrated a significant difference among the groups in terms of the distance moved [F (14, 128) =2.03, p=0.02]. The distance moved in block 1 was significantly decreased in the BDL+GA 30 mg/kg group compared with the BDL group (p=0.006). 

In block 2, a significant difference was observed in the distance moved by the BDL group compared with the sham group (p<0.0001), the BDL+GA 30 mg/kg group compared with the BDL group (p<0.0001), the BDL+CC group compared with the sham+CC group (p=0.013) and the BDL+ GA 30 mg/kg +CC group compared with the BDL+CC group (p=0.034). The distance moved in block 3 was significantly different in the BDL group compared with the sham group (p=0.001) and the BDL+CC group compared with the sham+CC group (p=0.001) (Figure 3A). 

In Figure 3B, the duration of finding the platform by the rats in different groups is shown. No interaction of group × training blocks was found in the groups [F (14,128) = 1.449, (p=0.14)] (Figure 3B). 

Two-way ANOVA did not show a significant difference among the groups in percent time of target quadrant [F (14, 129) =1.376, p=0.17] (Figure 3C). 

In the probe trail, one-way ANOVA presented a significant difference in the percent distance moved of target quadrant between the groups [F (7, 51) =47.36, p<0.0001]. In the BDL group, a significant decrease in the percent distance moved of target quadrant was observed compared with the sham group (p<0.0001). In the BDL+GA 30 mg/kg group, a significant increase in the percent distance moved of target quadrant was observed compared with the BDL group (p<0.0001). In the sham+CC group, a significant increase in the percent distance moved of target quadrant was observed compared with the sham group (p<0.0001). The BDL+CC group had a significant decrease in the percent distance moved of target quadrant compared with the sham+CC group (p<0.0001) and the BDL+GA 20 mg/kg +CC group showed a significant increase in the percent distance moved of target quadrant compared with the BDL+CC group (p=0.001). The BDL+ GA 30 mg/kg +CC group showed a significant decrease in the percent distance moved of target quadrant compared with the BDL+GA 30 mg/kg group (p<0.0001) (Figure 3D). 

In the probe trail, the percentage of time in the target quarter was significantly different among the groups [F (7, 47) =8.80, p<0.0001]. In the sham+CC group, a significant increase was observed compared with the BDL+CC group (p<0.0001). The BDL+GA 30 mg/kg group had a significant increase compared with the BDL+GA 30 mg/kg+CC group (p=0.013) (Figure 3E). 

## Discussion

In general, in our study, the results of the NOR test and the MVM test parameters showed that the BDL group had cognitive and memory impairments. Also, the groups receiving dorsomorphin demonstrated more cognitive impairment than the groups that did not receive dorsomorphin. Results of open field test parameters demonstrated an increase in anxiety-like behaviors in BDL groups and dorsomorphin-receiving groups. The BDL and dorsomorphin-receiving groups, which also received GA, demonstrated improved memory function and reduced anxiety-like behaviors. HE is a reversible neurological complication that occurs in both acute and chronic forms. This disease is caused by increased inflammation and oxidative stress in the brain and is associated with clinical symptoms such as dysfunction of the nervous system, sensory disorders, mental disorders, and memory and learning problems as well as poor concentration (Jiang et al., 2019b ▶). AMPK regenerates cellular energy sources during the metabolic damage. The beneficial effects of AMPK activation on preservation of neurons and reduction of apoptosis of these cells in hepatic encephalopathy using cannabinoid and sestrin2 compounds have been demonstrated in previous studies (Dagon et al., 2007 ▶; Hu et al., 2018 ▶). 

In general, the results of this study showed that BDL-induced encephalopathy affects the short-term memory of these animals in the form of failure to discriminate the novel object (NOR test). This finding was in line with the results of another study in this regard (Dhanda et al., 2018 ▶). In this investigation, it was found that dorsomorphin can affect cognitive skills and memory by inhibiting AMPK in the hippocampus, which has also been reported in previous works (Jiang et al., 2019a ▶; Rashtiani et al., 2021a ▶). In our study, GA at the doses of 20 and 30 mg/kg improved memory and learning performance, and reduced anxiety-like behaviors compared with the HE groups. This improvement was seen even in the dorsomorphin groups. In the metabolic syndrome, GA administration at the dose of 20 mg/kg for 60 days reduced hippocampal neurodegeneration and synaptic flexibility, resulting in improved nerve cell function and positive effects on short- and long-term memory (Diaz et al., 2020 ▶). In arsenic-induced memory and anxiety disorders, GA administration for four weeks at the doses of 50 and 100 mg/kg was able to improve memory and behavioral disorders (Samad et al., 2019 ▶). GA has been shown to be effective in neuronal plasticity and activating AMPK due to its antioxidant activities (Diaz et al., 2020 ▶; Doan et al., 2015 ▶). 

General motor activity was determined by the total distance moved by the rats and the level of anxiety was assessed using the time spent in the center (Cho et al., 2020 ▶). Rearing behavior indicates good motor activity and low stress in the animal, and grooming behavior shows stress and anxiety (Rojas-Carvajal and Brenes, 2020 ▶; Sturman et al., 2018 ▶). The findings revealed that HE decreased rearing behavior and increased grooming behavior (Hajipour et al., 2021 ▶). Our study also showed that HE and inhibition of AMPK increased anxiety-like behaviors. Similar to the results of the present study, evidence shows that GA is involved in alleviating anxiety-like behavior and increasing motor activity (Salehi et al., 2018 ▶). GA may reduce anxiety-like behaviors and improve motor activity by activating AMPK. 

The impairment of spatial memory and learning a well as cognitive disorders can be considered diagnostic factors for HE (Dhanda et al., 2018 ▶). In this study, during the acquisition, the memory and learning levels of the rats with encephalopathy and inhibited AMPK were reduced, and the distance moved and time spent on finding the platform were increased. The longer it takes to find the platform, the lower the learning and memory would be. 

In general, the sham rats spent more time than the other rats in the target quadrant searching for the platform. On the other hand, the rats with encephalopathy, in which AMPK was inactivated, had shorter duration in the target quadrant, indicating short-term memory impairment in the rats. Rats usually spend more time on finding the platform in the first test. But, after several tests, those with good memory find the platform in less time and with the shortest distance moved. Rats with HE that had inactivated AMPK spent more time on finding the platform and had a lower percentage of presence in the target quarter, indicating that their memory and learning were impaired due to hippocampal damage (Lu et al., 2020b ▶). In this study, spatial memory impairment was seen in BDL rats and the rats receiving dorsomorphin (except for the sham+CC group), and was indicated by the evident increase in time spent on the average latency and decrease in the percent of time spent on the target quadrant in probe trail. The present findings were compatible with those of other studies (Aghaei et al., 2017 ▶; Li et al., 2017 ▶). In this study, GA produced positive signals for improving memory and learning. It may improve learning and enhance memory by reducing apoptosis and inflammation in the liver cells and by activating AMPK in the liver and hippocampus (Tanaka et al., 2020 ▶). According to the present results, administration of dorsomorphin to HE rats led to cognitive deficit, and learning and memory weakness as well as higher levels of anxiety. However, the sham group that received dorsomorphin showed none of these complications and, in some cases, performed better than the other groups. It was demonstrated that dorsomorphin reduced memory and learning in rats/mice by inactivating AMPK, but co-administering ghrelin was effective in improving memory and learning (Zahiri et al., 2019 ▶). In a diabetic model, adiponectin improved memory by activating AMPK (Rashtiani et al., 2021a ▶). *In vitro* studies showed that the effect of dorsomorphin as an AMPK inhibitor was dependent on its concentration. Low concentration of dorsomorphin did not reduce the amount of AMPK, but its high concentration, even when combined with lipopolysaccharide (an activator of AMPK), decreased the amount of AMPK (Łabuzek et al., 2010 ▶). Oxidative stress caused an increase in AMPK which resulted in the reduction of stress, apoptosis and cellular damage (Kosuru et al., 2018 ▶). In the present study, dorsomorphin as an inhibitor of AMPK might have exerted its effects in the BDL groups due to oxidative stress, cellular damage and decreased intracellular ATP; however, in the sham group which received a similar dose of dorsomorphin, AMPK was not inhibited and, as a result, memory loss and cognitive impairment were not observed. In order to prevent cognitive impairment, it is necessary to increase the induction of the AMPK pathway (Huang et al., 2019 ▶; Łabuzek et al., 2010 ▶). In this study, GA with its antioxidant properties reduced the effect of dorsomorphin, thus increasing the activity of AMPK; by increasing the ATP of neurons, memory impairments and anxiety-like behaviors were reduced in the BDL rat model. 

In this study, there was evidence that HE impaired learning, memory and spatial memory in male rats. Moreover, it led to increased anxiety and movement disorders. However, dorsomorphin caused cognitive disorders and anxiety-like behaviors in BDL-induced groups. GA was effective in improving cognitive and memory disorders as well as anxiety-like behaviors probably due to activating AMPK. Further studies focusing on inflammatory cytokines, oxidative stress and AMPK activation pathways are needed. 

## Conflicts of interest

The authors have declared that there is no conflict of interest.
